# Genotyping both live and dead animals to improve post-weaning survival of pigs in breeding programs

**DOI:** 10.1186/s12711-024-00932-4

**Published:** 2024-09-18

**Authors:** Md Sharif-Islam, Julius H. J. van der Werf, Mark Henryon, Thinh Tuan Chu, Benjamin J. Wood, Susanne Hermesch

**Affiliations:** 1 AGBU, a joint venture of NSW Department of Primary Industries and University of New England, Armidale, NSW 2351 Australia; 2 School of Environmental and Rural Science, University of New England, Armidale, 2351 Australia; 3 Danish Pig Research Center, Danish Agriculture and Food Council, Axeltorv 3, 1609 Copenhagen V, Denmark; 4 Center for Quantitative Genetics and Genomics, Aarhus University, 8000 Aarhus, Denmark; 5 School of Veterinary Science, University of Queensland, Brisbane, QLD 4072 Australia

## Abstract

**Background:**

In this study, we tested whether genotyping both live and dead animals (GSD) realises more genetic gain for post-weaning survival (PWS) in pigs compared to genotyping only live animals (GOS).

**Methods:**

Stochastic simulation was used to estimate the rate of genetic gain realised by GSD and GOS at a 0.01 rate of pedigree-based inbreeding in three breeding schemes, which differed in PWS (95%, 90% and 50%) and litter size (6 and 10). Pedigree-based selection was conducted as a point of reference. Variance components were estimated and then estimated breeding values (EBV) were obtained in each breeding scheme using a linear or a threshold model. Selection was for a single trait, i.e. PWS with a heritability of 0.02 on the observed scale. The trait was simulated on the underlying scale and was recorded as binary (0/1). Selection candidates were genotyped and phenotyped before selection, with only live candidates eligible for selection. Genotyping strategies differed in the proportion of live and dead animals genotyped, but the phenotypes of all animals were used for predicting EBV of the selection candidates.

**Results:**

Based on a 0.01 rate of pedigree-based inbreeding, GSD realised 14 to 33% more genetic gain than GOS for all breeding schemes depending on PWS and litter size. GSD increased the prediction accuracy of EBV for PWS by at least 14% compared to GOS. The use of a linear versus a threshold model did not have an impact on genetic gain for PWS regardless of the genotyping strategy and the bias of the EBV did not differ significantly among genotyping strategies.

**Conclusions:**

Genotyping both dead and live animals was more informative than genotyping only live animals to predict the EBV for PWS of selection candidates, but with marginal increases in genetic gain when the proportion of dead animals genotyped was 60% or greater. Therefore, it would be worthwhile to use genomic information on both live and more than 20% dead animals to compute EBV for the genetic improvement of PWS under the assumption that dead animals reflect increased liability on the underlying scale.

## Background

Post-weaning survival (PWS) is an economically important trait for growing pigs. It is also a proxy for animal welfare [1]. The death of a pig after weaning results in a loss of profit that is proportional to the expenditure associated with feed, labour and capital. Genetic gain for PWS has increased with the advent of genomic selection. The accuracy of estimated breeding values (EBV) for PWS has increased by 20 to 50% with the use of genomic information based on cross-validation [2, 3]. A common challenge with using genomic information to predict EBV for PWS is that only surviving pigs are genotyped [2], because dead pigs are not candidates for selection. In the case of selective genotyping strategies [4], an obvious research question is: which individuals should be genotyped? Genotyping only surviving pigs refers to a scenario in which only the phenotypically top animals are genotyped, whereas genotyping both live and dead animals refers to a scenario in which phenotypically contrasting animals are genotyped. Genotyping both surviving and dead individuals can increase the power to detect single nucleotide polymorphisms (SNPs) or quantitative trait loci (QTL) associated with PWS [5, 6]. Certainly, genotyping phenotypically contrasting animals can increase the accuracy of genomic selection, increase genetic gain, and decrease the bias of EBV for a continuous trait compared to genotyping only the top animals, which is found in simulation studies [7, 8]. These studies did not consider a binary trait such as a survival trait. We are generally supported by Liu et al. [9] who found that genotyping all animals resulted in a 2 to 6% higher accuracy of genomic selection than genotyping only live animals. Thus, genotyping both live and dead animals could differentiate between SNPs that are associated with survival or death, which would allow a more accurate ranking of animals based on genetic merit for PWS. Based on this background, we reasoned that genotyping both live and dead animals realises more genetic gain for PWS than genotyping only live animals at the same rate of inbreeding. We tested this hypothesis by (1) including the genotypes of dead animals in genetic evaluation of PWS on genetic gain in pig breeding programs, (2) investigating the optimum proportion of dead animals to genotype, and (3) comparing the genetic gain using different proportions of genotyped dead and live animals with genetic evaluation using a threshold or a linear model.

## Methods

### Design

We used stochastic simulation to estimate rates of genetic gain $(\Delta \text{G})$ realised for PWS at 1% rate of pedigree inbreeding $(\Delta F)$ in optimum-contribution selection (OCS) when we genotyped both live and dead animals and when we genotyped only live animals. We did this by simulating three genotyping strategies:

i) Increasing the number of animals genotyped by increasing the number of dead animals genotyped. All (100%) live animals and 0, 20, 40, 60, 80, or 100% dead animals were genotyped.
ii) Fixed number of animals genotyped by varying the proportion of live and dead animals genotyped. All live animals were genotyped excluding the equivalent number of dead animals genotyped. That is, 0, 20, 40, 60, 80, or 100% dead animals were randomly chosen to be genotyped and an equivalent number of live animals were randomly chosen not to be genotyped.
iii) Increasing number of live animals genotyped; no dead animals genotyped: 0, 20, 40, 60, 80, or 100% of live animals were genotyped. Live animals were chosen randomly.

These genotyping strategies were carried out in breeding schemes with three initial PWS (50, 90, and 95%), two litter sizes (six and 10), and two EBV prediction models (threshold and linear models with genomic and pedigree information). Pedigree selection was simulated using pedigree-based EBV as a reference for two prediction methods. PWS had a heritability of 0.02 on the observed scale. The trait was controlled by 7702 biallelic QTL (quantitative trait loci). The QTL were randomly distributed across a 30 M genome that consisted of 18 167-cM chromosome pairs. The genome contained 54,218 biallelic markers that were used to calculate genomic EBV. The number of chromosomes and LD between alleles at the markers were simulated to resemble those in three commercial breeds of Danish pigs [10]. Breeding schemes were run for 10 discrete generations ( t = 1 … 10). Animals in the base populations were randomly selected in generation t = 1. In generations t = 2 … 10, selection candidates were allocated matings by OCS. All animals were genotyped before selection in generations t = 2 … 10. Each combination of genotyping strategy, initial PWS, litter size, and prediction model was replicated 50 times.

For each replicate, variance components were estimated on the simulated data with both the threshold and linear models with no selection in each replicate. The breeding scenarios varying in litter size and an initial PWS for estimating variance components were run for six discrete generations where live animals were randomly selected from generations $\text{t}$ = 1 to 6, without use of OCS to control inbreeding. Pedigree information was used to estimate variance components because variance component estimates can be biased if genomic information differs between selective genotyping strategies [9, 11]. Estimated variance components were used for calculating EBV with both the threshold and linear models.

### Breeding schemes

In each breeding scheme, three hundred matings were allocated to either 1800 selection candidates—900 males and 900 females or 3000 selection candidates—1500 males and 1500 females—by OCS in generations t = 2 to 10. Three hundred females were allocated a single mating and males were allocated 0, 1, 2…, or 40 matings. No restriction was imposed on the number of males used as parents. The 300 dams selected by OCS were paired randomly to the selected sires. Each pair (dam) produced either six or 10 offspring, resulting in 20 full sib families and 1800 offspring or 3000 offspring. Offspring were assigned as males or females with a probability of 0.5. The PWS rate in generation t = l was set to 90, 95, or 50% and litter size was set to six or 10, resulting in breeding schemes that will be referred to as LS6S90, LS6S95, LS650 and LS10S90.

### Simulation procedure

#### Generations − 1000 to − 1: founder population

The simulation of the pig genome in the founder population is described in [12]. Linkage disequilibrium (LD) between the 54,218 markers and 7702 QTL was established in a founder population of 25 males and 25 females using a Fisher-Wright inheritance model [13, 14].

The 54,218 markers and 7702 QTL in our simulated breeding scenarios were all segregating in generation $t$ = − 1 of the founder population. The additive-genetic effects of the bi-allelic alleles at the 7702 segregating QTL were standardised so that the total additive-genetic variance on the underlying scale for the trait under selection was equal to 0.03, 0.06 and 0.09 for PWS of 50, 90, 95% in founder population. No new mutations were generated after the founder population was simulated. Chromosomes from the 25 males and 25 females in generation $t$ = − 1 of the founder population were pooled: 18 pools of 100 chromosomes. Each pool consisted of 50 chromosome pairs of the $i$th chromosome ($i$ = 1 … 18) from 50 founder animals. The breeding scenarios were initiated by sampling base populations from these chromosome pools.

#### Generation 0: base populations

Each replicate combination of genotyping strategy, litter size and prediction method was initiated by sampling a unique base population. Twenty males and 300 females were sampled in the simulated breeding scheme. The genotype of each base animal was sampled from the 18 pools of chromosomes in generation $t$ = − 1 of the founder population. For chromosome $i$ ($i$ = 1 … 18), two chromosomes were randomly sampled without replacement from the $i$ th pool of 100 chromosomes. The sampled chromosomes were replaced before the next base animal was sampled. Base animals were assumed to be unrelated and non-inbred based on pedigree and IBD alleles. They were genotyped, but not phenotyped for the trait under selection.

#### Generation 1: Random selection in base populations

In the simulated breeding scheme, 20 sires and 300 dams were selected. Each sire was mated with 15 dam. Each dam produced six or 10 offspring.

#### Generations 2 to 10: optimum-contribution selection

Animals were selected based on best linear unbiased prediction (BLUP) of EBVs using pedigree or genomic information in generations $t$ = 2 … 10. Residual maximum lilkelihood (REML) estimates of variance components on the simulated data were used in each BLUP run across the generations. Animals were allocated matings by OCS in generations $t$ = 2 … 10.

### PWS

PWS was assessed as a binary trait and assumed to follow a threshold-liability model [15]. PWS of the *i*th pig was PWS_*i*_ = 0 (died) if U_*i*_ ≤ *t* and PWS_*i*_ = 1 (survived) if U_*i*_ > *t*, where U_*i*_ is the pig’s unobserved underlying liability and *t* is a fixed threshold set to attain the predefined average PWS (50, 90, or 95%). The unobserved underlying liability of pig *i* was:

1 $${\text{U}}_{\text{i}}= {\text{a}}_{\text{i}}+ {\text{c}}_{\text{i}}+ {\text{e}}_{\text{i}},$$

where ${\text{a}}_{\text{i}}$ is the TBV of the animals, calculated as the sum of additive genetic effects for the 7702 QTL, ${\text{c}}_{\text{i}}$ is a litter effect sampled from ${\text{c}}_{\text{i}}\sim N(0,{\upsigma }_{\text{c}}^{2})$, and ${\text{e}}_{\text{i}}$ is the residual value sampled from ${\text{e}}_{\text{i}}\sim N(0, {\upsigma }_{\text{e}}^{2})$. For PWS equal to 50, 90, and 95%, ${\upsigma }_{\text{c}}^{2}$ was set to 0.06, 0.15, and 0.22, and ${\upsigma }_{\text{e}}^{2}$ was set to 0.91, 0.79, and 0.69 to obtain an heritability on the observed scale of ${\text{h}}_{\text{o}}^{2}$ of 0.02.

The heritability on the observed-scaled (${\text{h}}_{\text{o}}^{2}$) of 0.02 can be transformed to heritability on the underlying scale using the formula of Dempster and Lerner [16]:

2 $${\text{h}}_{\text{l}}^{2}=\frac{{\text{h}}_{\text{o}}^{2}\text{K}(1-\text{K})}{{\text{z}}^{2}},$$

where $\text{K}$ is the percentage of PWS which, in this study, was assumed to be 50%, 90% and 95%, $\text{z}$ is the height of the normal distribution curve at the threshold, ${\text{h}}_{\text{l}}^{2}$ is the heritability on the liability scale and ${\text{h}}_{\text{o}}^{2}$ is the observed-scale heritability. For PWS equal to 50, 90, and 95%, the heritability of PWS on the unobserved liability scale was ${\text{h}}_{\text{l}}^{2}$ = 0.03, 0.06, and 0.09, respectively.

### Estimating variance components for PWS

The REML estimates of variance components for PWS were calculated using both animal and sire models with the threshold and linear methods on the simulated data. The linear model assumes normality of the binary trait, while the threshold model assumes that the observed binary responses are the result of underlying normally distributed latent variables [17]. Preliminary results showed that variance components estimated with the sire model were the most consistent with the true heritability (0.02, on the observed scale). Therefore, in this study, variance components derived from the sire model were used to predict EBV under the animal model in each replicate for PWS.

PWS assessed as a binary trait was assumed to follow a threshold liability model [15], which postulates that there is an unobserved underlying variable (i.e., liability) for the ${i}^{th}$ animal, ${U}_{i}$, where ${Y}_{i}=0$ (died) if ${U}_{i}\le t$, ${Y}_{i}=1$ (survived) if ${U}_{i}>t$, and $t$ is a fixed threshold set to zero.

3 $$\mathbf{U}={\mathbf{X}\mathbf{b}+\mathbf{Z}}_\mathbf{1}\mathbf{s}+ {\mathbf{Z}}_\mathbf{2}\mathbf{c}+\mathbf{e},$$

$$f(y=0)$$ if $${U}_{i}\le t$$,

$f(y=1)$ if ${U}_{i}>t$, where $\mathbf{U}$ is the vector of underlying liabilities to survive and the estimated variance components from this model were on the underlying scale, $\mathbf{b}$ is the vector of fixed generation effects, $\mathbf{s}$ is the vector of the sire genetic effect, $\mathbf{c}$ is the random litter effect and $\mathbf{e}$ is the vector of the residual effect. The distribution of the random effects was as follows:

$$\left(\begin{array}{c}\mathbf{s}\\ \mathbf{c}\\ \mathbf{e}\end{array}\right) \sim \text{ N }\left(\left[\begin{array}{c}0\\ 0\\ 0\end{array}\right], \left[\begin{array}{ccc}{{\mathbf{A}}_{\mathbf{s}}\upsigma }_{\text{s}}^{2}& 0& 0\\ 0& {\mathbf{I}\upsigma }_{{\text{c}}_{\text{s}}}^{2}& 0\\ 0& 0& {\mathbf{I}\upsigma }_{{\text{e}}_{\text{s}}}^{2}\end{array}\right]\right),$$

where, ${\mathbf{A}}_{\mathbf{s}}$ is the pedigree relationship among sires, ${\upsigma }_{\text{s}}^{2}$ is the sire genetic variance, and ${\upsigma }_{{\text{c}}_{\text{s}}}^{2}\text{ and}$ ${\upsigma }_{{\text{e}}_{\text{s}}}^{2}$ are the litter variance and residual variance in the threshold sire model. A linear sire model was also used to estimate the variance components in the simulated data where the observations of PWS were treated as a normally distributed trait.

### Estimating breeding values for PWS

Breeding values for PWS were estimated using both linear and threshold animal models similar to Eq. (3) and the pedigree relationships among sires in Eq. (3) were replaced with pedigree relationships among all animals. The same variance components from the sire linear and threshold models were used to predict EBV for all generations within a replicate (Table 1). The genetic variance for calculating EBV was set to four times the estimated sire variance, the litter variance was adjusted by subtracting the estimated sire variance.The single-step genomic breeding value was estimated using the $\mathbf{H}$ matrix instead of $\mathbf{A}$ [18].

**Table 1:** Estimated variance components (VC) for post-weaning survival using animal and sire linear and threshold models in the simulated data

Breeding schemes	VC	Linear model		Threshold model	
		Animal	Sire	Animal	Sire
LS6S90	${\upsigma }_{\text{a}}^{2}\text{ or }{\upsigma }_{\text{s}}^{2}$	0.0018 (0.001)	0.0004 (0.0002)	0.0318 (0.02)	0.0154 (0.008)
	${\upsigma }_{\text{c}}^{2}$	0.0052 (0.001)	0.0058 (0.0010)	0.1048 (0.02)	0.1169 (0.017)
	${\upsigma }_{\text{e}}^{2}$	0.0809 (0.004)	0.0814 (0.0060)	1	1
	h^2^	0.02	0.02	0.03	0.05
LS6S50	${\upsigma }_{\text{a}}^{2}\text{ or }{\upsigma }_{\text{s}}^{2}$		0.0011 (0.0005)		0.0068 (0.003)
	${\upsigma }_{\text{c}}^{2}$		0.0136 (0.0019)		0.0794 (0.012)
	${\upsigma }_{\text{e}}^{2}$		0.2345 (0.0020)		1
	h^2^		0.02		0.03
LS10S90	${\upsigma }_{\text{a}}^{2}\text{ or }{\upsigma }_{\text{s}}^{2}$				0.0151 (0.005)
	${\upsigma }_{\text{c}}^{2}$				0.1378 (0.013)
	${\upsigma }_{\text{e}}^{2}$				1
	h^2^				0.05
LS6S95	${\upsigma }_{\text{a}}^{2}\text{ or }{\upsigma }_{\text{s}}^{2}$				0.0211 (0.011)
	${\upsigma }_{\text{c}}^{2}$				0.1411 (0.022)
	${\upsigma }_{\text{e}}^{2}$				1
	h^2^				0.07

${\upsigma }_{\text{a}}^{2}$ = additive genetic variance, ${\upsigma }_{\text{s}}^{2}$ = sire genetic variance, ${\upsigma }_{\text{c}}^{2}$ = common environmental variance, ${\upsigma }_{\text{e}}^{2}$= residual variance, h^2^ = heritability (${\upsigma }_{\text{a}}^{2}/ {\upsigma }_{\text{a}}^{2}+{\upsigma }_{\text{c}}^{2}$+${\upsigma }_{\text{e}}^{2}$) or $({4\upsigma }_{\text{s}}^{2}/ {\upsigma }_{\text{s}}^{2}+{\upsigma }_{\text{c}}^{2}$+${\upsigma }_{\text{e}}^{2}$). Results are the mean of 50 replicates. Standard error is given in parenthesis. h^2^ from the linear model is expressed on the observed scale and from the threshold model it is expressed on the underlying scale

For the threshold model, dstimated breeding values were transformed to the probability scale using the equation from Hidalgo et al. [19]:

$${\text{P}}_{\text{i}}=1-\text{ \O }\left(\frac{{\upmu }_{\text{i}}-{\upmu }_{\text{u}}}{{\upsigma }_{\text{u}}}\right),$$

where ${\text{P}}_{\text{i}}$ is the probability of survival for animal i, Ø is the standard cumulative distribution function, ${\upmu }_{\text{i}}$ is the estimated breeding value of animal $\text{i}$, ${\upmu }_{\text{u}}$ is the mean estimated breeding value of all animals, ${\upsigma }_{\text{u}}$ is the standard deviation of estimated breeding values of all animals.

### Criteria used for to assess scenarios

The rate of true genetic gain (${\Delta \text{G}}_{\text{true}}$) on the underlying scale was calculated for each scenario and accuracy and bias of EBV of live animals were used to investigate the mechanisms that underlie the differences between scenarios.

${\Delta \text{G}}_{\text{true}}$ was calculated for each replicate as a linear regression of ${\text{G}}_{\text{t}}$ on time $\text{t}$, where ${\text{G}}_{\text{t}}$ is the average TBV of the animals born in generations $\text{t}$ = 5 to 10 on the underlying scale. The accuracy and bias of the EBV of live animals were calculated in each replicate as the correlation and regression of TBV with and on EBV of live animals, with EBV expressed on the probability scale. Accuracy and bias of EBV were compared between the genotyping strategies for both the linear and the threshold models. All results were presented as the mean of 50 replicates.

### Software

Breeding programs were simulated using the ADAM program [20], the OCS was run using the EVA program [21]. Variance componenets and EBVs were calculated using the DMU software [22].

## Results

The main objective of this study was to compare genetic gain realised by different genotyping strategies with different combinations of breeding schemes and prediction models.

### Rate of genetic gain

Genotyping both live and dead animals for selection realised more genetic gain than genotyping only live animals for all breeding schemes. Genetic gain was compared at a rate of 1% pedigree inbreeding for all genotyping strategies. When the proportion of genotyped dead pigs increased from 20 to 100%, genotyping both live and dead animals realised 14 to 33% more Δ ${\text{G}}_{\text{true}}$ than genotyping live animals only in each of the PWS scenarios (Tables 2, 3, and 4). The genetic gain realised from genotyping all live and 20% of the dead animals was only 10% higher than the Δ ${\text{G}}_{\text{true}}$ realised by genotyping only live animals. Genotyping 60 to 80% of the dead animals and all live animals realised a larger (21%) Δ ${\text{G}}_{\text{true}}$ than genotyping only live animals. Breeding schemes LS6S90, LS6S95 and LS6S50 differed in PWS, i.e. 90, 95 and 50%, respectively. The genetic variance on the underlying scale differed in these scenarios with the different simulated PWS but the phenotypic variance on the underlying scale was 1 for all scenarios. All live animals were genotyped in each PWS scenario but both the number of live animals and the total number of genotyped animals differed. Consequently, the genetic gains from the PWS scenarios were not directly comparable. However, when Δ ${\text{G}}_{\text{true}}$ was expressed in genetic standard deviation using, the breeding scheme LS6S50 had the highest Δ ${\text{G}}_{\text{true}}$ (Table 5). Genetic gain in PWS benefited from genotyping both live and dead animals compared to genotyping only live animals in the three breeding schemes (Table 5).

**Table 2:** Rate of genetic gain (expressed on the underlying scale) for post-weaning survival in the LS6S90 and LS6S50 breeding schemes

Scenario	Rate of genetic gain			
	Threshold model		Linear model	
	LS6S90	LS6S50	LS6S90	LS6S50
Genotyping live animals only	0.093 (0.001)	0.081 (0.001)	0.098 (0.001)	0.084 (0.001)
Genotyping live + 20% dead animals	0.103 (0.001)	0.089 (0.001)	0.104 (0.001)	0.082 (0.001)
Genotyping live + 40% dead animals	0.108 (0.001)	0.086 (0.001)	0.112 (0.002)	0.087 (0.001)
Genotyping live + 60% dead animals	0.113 (0.001)	0.089 (0.001)	0.117 (0.002)	0.088 (0.001)
Genotyping live + 80% dead animals	0.114 (0.001)	0.089 (0.001)	0.117 (0.002)	0.092 (0.001)
Genotyping live + 100% dead animals	0.118 (0.001)	0.093 (0.001)	0.115 (0.001)	0.091 (0.001)

Results are the mean of 50 replicates. Standard error is shown in parenthesis

**Table 3:** Rate of genetic gain (expressed on the underlying scale) for post-weaning survival in the LS6S90 breeding scheme

Scenario	Rate of genetic gain
Genotyping 1620 live (all) animals	0.093 (0.001)
Genotyping 1584 live + 36 dead animals	0.105 (0.002)
Genotyping 1548 live + 72 dead animals	0.102 (0.002)
Genotyping 1512 live + 108 dead animals	0.112 (0.002)
Genotyping 1476 live + 144 dead animals	0.114 (0.002)

Standard error is shown in parenthesis. Results are the mean of 50 replicates

**Table 4:** Rate of genetic gain (expressed on the underlying scale) for post-weaning survival in the LS10S90 and LS6S95 breeding schemes

Scenario	Rate of genetic gain	
	LS10S90	LS6S95
Pedigree-based selection	0.064 (0.001)	0.054 (0.002)
Genotyping all live animals	0.108 (0.003)	0.102 (0.002)
Genotyping all live + 60% dead animals	0.140 (0.003)	0.118 (0.002)

Standard error is shown in parenthesis. Results are the mean of 50 replicates

**Table 5:** Rate of genetic gain (expressed in genetic standard deviation) for post-weaning survival in the LS6S50, LS6S90 and LS6S95 breeding schemes

Scenario	Rate of genetic gain		
	LS6S50	LS6S90	LS6S95
Pedigree selection	0.31 (0.01)	0.23 (0.01)	0.18 (0.01)
Genotyping all live animals	0.50 (0.01)	0.38 (0.01)	0.34 (0.01)
Genotyping all live + 60% dead animals	0.54 (0.01)	0.46 (0.01)	0.39 (0.01)

Standard error is shown in parenthesis. Results are the mean of 50 replicates

### Impact of average litter size

Genetic gain was compared for breeding scenarios that differed in litter size. A larger litter sizes enabled a higher selection intensity. Genotyping both live and dead animals realised more genetic gain than genotyping only live animals when litter size was larger. Genotyping both live and dead animals realised 6 to 7% more Δ ${\text{G}}_{\text{true}}$ for PWS in the LS10S90 breeding scheme compared to the LS6S90 breeding scheme (Tables 2 and 3).

### Prediction models

Genotyping strategies for PWS were compared using two prediction methods, i.e. using the linear or threshold model. Genetic gain realised by genotyping both live and dead animals was not affected by the prediction method used, for all breeding schemes (Table 2). Within each prediction model, Δ ${\text{G}}_{\text{true}}$ for PWS benefited from genotyping both live and dead animals, which supports the hypothesis of this study.

Pedigree-based selection was conducted as a point of reference in this study. In the LS6S90 breeding scheme, genotyping only the live animals realised a 52% higher ${\Delta \text{G}}_{\text{true}}$ for PWS compared to pedigree-based selection. Genotyping 20 to 100% of the live animals per generation realised a 16 to 52% higher Δ ${\text{G}}_{\text{true}}$ compared to the genetic gain achieved in pedigree-based selection (Table 6).

**Table 6:** Rate of genetic gain, accuracy and bias of EBV for post-weaning survival in the LS6S90 breeding scheme using the threshold model

Scenario	Rate of genetic gain	Accuracy of EBV	Bias of EBV
Pedigree-based selection	0.061 (0.001)	0.18 (0.01)	0.55 (0.03)
Genotyping 20% live pigs	0.071 (0.002)	0.23 (0.01)	0.65 (0.04)
Genotyping 40% live pigs	0.077 (0.002)	0.25 (0.01)	0.68 (0.04)
Genotyping 60% live pigs	0.086 (0.002)	0.26 (0.01)	0.69 (0.03)
Genotyping 80% live pigs	0.088 (0.002)	0.26 (0.01)	0.71 (0.03)
Genotyping all live pigs	0.093 (0.003)	0.27 (0.01)	0.73 (0.04)

Standard error is shown in parenthesis. Results are the mean of 50 replicates

### Improvement in post-weaning survival

The average PWS in generation 10 (Table 7) when genotyping only the live animals was 1% higher than with pedigree-based selection in the LS6S90 breeding scheme and 9% higher for the LS6S50 breeding scheme. Furthermore, it was 2% higher when genotyping the live and 40% of the dead animals than with pedigree-based selection and 1% higher than genotyping only the live animals for the LS6S90 breeding scheme.

**Table 7:** Average post-weaning survival in generation 10 in the LS6S90 and LS6S50 breeding schemes using the threshold model

Scenario	Average post-weaning survival in generation 10	
	**LS6S90**	**LS6S50**
Pedigree-based selection	0.96 (0.001)	0.66 (0.004)
Genotyping all live pigs	0.97 (0.001)	0.73 (0.003)
Genotyping all live + 40% dead pigs	0.98 (0.001)	0.75 (0.004)
Genotyping all live + 60% dead pigs	0.98 (0.001)	0.74 (0.004)
Genotyping all live and dead pigs	0.98 (0.001)	0.75 (0.004)

Standard error is shown in parenthesis. Results are the mean of 50 replicates

#### Accuracy and bias

Genotyping both live and dead animals increased the accuracy of EBV for live animals compared to genotyping only live animals (Table 8). The regression of TBV on EBV of live animals was less than 1 in the scenarios in which different proportions of dead animals were genotyped, along with all live animals, but the deviation from 1 decreased as the proportion of genotyped dead animals increased (Table 8). The accuracy of EBV when genotyping all live and 20 to 80% of the dead animals was 6 to 12% higher than when genotyping only the live animals. Using both the linear and the threshold model showed a similar trend in the increase in prediction accuracy of EBV with the genomic information from both live and dead aniamls.

**Table 8:** Accuracy and bias of EBV for post-weaning survival in live animals in the LS6S90 breeding scheme

Scenario	Accuracy of EBV		Bias of EBV	
	Threshold model	Linear model	Threshold model	Linear model
Pedigree-based selection	0.18 (0.01)	0.19 (0.01)	0.55 (0.01)	0.58 (0.01)
Genotyping live pigs only	0.27 (0.01)	0.28 (0.01)	0.73 (0.01)	0.77 (0.01)
Genotyping live + 20% dead pigs	0.29 (0.01)	0.30 (0.01)	0.75 (0.01)	0.80 (0.01)
Genotyping live + 40% dead pigs	0.30 (0.01)	0.31 (0.01)	0.78 (0.01)	0.80 (0.01)
Genotyping live + 80% dead pigs	0.31 (0.01)	0.32 (0.01)	0.79 (0.01)	0.81 (0.01)

Results are the mean of 50 replicates. Standard error is shown in parenthesis

## Discussion

Our findings support the hypothesis that genotyping both live and dead pigs results in more genetic gain for PWS than genotyping only live animals with genomic selection, under the assumption that the underlying liability for mortality has a genetic basis. Our findings also indicate that at least 60% of the dead animals need to be genotyped for an economically viable genotyping strategy given that the phenotypes for litter size and mortality of all the animals are available for prediction. Genotyping all live and 60% of randomly selected dead animals resulted in nearly as much genetic gain as genotyping all live and dead animals. Having phenotype records for all the live and dead animals and pedigree data is essential because breeders would not have to genotype all dead animals if all phenotype and pedigree data were included in the single-step genomic evaluation. This finding shows the benefits of a good genotyping strategy for PWS, which is important for the pig breeding industry to improve this trait by genomic selection. However, since all live animals were genotyped in all genotyping scenarios, the number of genotyped live animals was fixed. Some might argue that additional ${\Delta \text{G}}_{\text{true}}$ from genotyping dead animals might be due to genotyping more animals, not to genotyping dead animals. To rule out this possibility, we simulated genotyping scenarios in which 20 to 80% of randomly selected dead animals were genotyped and an equal number of genotypes from live animals was excluded. Hence, the difference in ${\Delta \text{G}}_{\text{true}}$ would be only due to genotyping dead versus live animals, with the same total number of animals genotyped. We found that genotyping both live and dead animals resulted in significantly higher ${\Delta \text{G}}_{\text{true}}$ than genotyping only live animals. Therefore, it is recommended to invest in recording phenotypes and pedigree data and in genotyping both dead and live animals for improvement of PWS by genomic selection.

The findings of this study have implications for both economic and welfare issues in pig production and it should also be considered that handling dead animals is stressful for industry staff. From an economic point of view, it can also be profitable to invest in genotyping dead animals, because the death of a weaned pig causes a big monetary loss due to investment in feed, labor, and capital for rearing pigs [23]. The economic implications of genotyping dead pigs are illustrated with a simple cost–benefit analysis by transforming the rate of genetic gain on the underlying scale to the observed binary scale using a cumulative distribution function. Based on the increase in the rate of genetic gain on the underlying scale from 0.102 for the scenario in which only live pigs are genotyped to 0.118 for the scenario in which both live and 60% of the dead pigs are genotyped in the LS6S95 breeding scheme, the PWS of growing pigs increased by 0.0013 (0.9597 to 0.9610) on the observed binary scale. Assuming the economic value of 182 A$ per growing pig for PWS [23], genotyping both live and 60% of the dead animals results in an additional economic gain of 0.23 A$ per growing pig. The breeding scheme was based on 1800 pigs and one round of selection leads to an additional 414 A$ for this nucleus population. The cost of genotyping 60% of the dead pigs (54 dead pigs in the LS6S95 breeding scheme) was 1350 A$ per round of selection assuming a genotyping cost of 25 A$ per pig. Genetic gain is permanent and this additional genetic gain indicates that it would take three rounds of selection to pay-off the investment in genotyping dead pigs in our simulation. These benefits ignore the additional benefits that are realised in the commercial population which depend on the size of the commercial population. A small commercial population of only 5900 pigs is required to pay-off the investment over just one round of selection with genotyping dead pigs in a fully integrated breeding scheme. These examples outline the economic implications of genotyping both dead and live animals which can be used by pig breeding companies to make their business decision regarding genotyping strategies for genetic improvement of PWS.

In this study, the additional genetic gain in PWS through genotyping both live and dead animals can be explained by the greater accuracy of EBV compared to accuracy when genotyping only live animals. Genotyping animals that are in an extreme category relevant to the population mean can increase the power to differentiate the effects of the SNPs associated with the trait [5, 6]. We found at least 14% greater accuracy of EBV of live animals when both live and dead animals were genotyped compared to the scenario in which only live animals were genotyped in the LS6S90 breeding scheme. Greater accuracy of EBV means more accurate ranking and selection of breeding animals for PWS. This increased accuracy of EBV translated into higher genetic gain since we found that genotyping both live and dead animals led to greater genetic gain for PWS than genotyping only live animals. Our results are supported by those of Liu et al. [9] who found that genotyping both live and live animals resulted in higher accuracy of EBV than genotyping only live animals. Several other studies reported an increase in accuracy of EBV for continuous traits for a given number of genotyped animals when phenotypically contrasting animals are genotyped, i.e. top and bottom selection candidates [7, 24] For example, Gowane et al. [7] found 8.3% greater accuracy of EBV when the top 25% and bottom 25% selection candidates for a continuous trait were genotyped compared to the scenario in which only the top 50% selection candidates were genotyped. Boligon et al. [24] also found a higher accuracy of the EBV and a lower predictive mean square error when phenotypically contrasting animals were genotyped compared to genotyping only top animals. This superiority of genotyping phenotypically contrasting animals is explained by the fact that the animals with extreme phenotypes are more informative for the regression line of true breeding values on estimated breeding values [25]. Therefore, in practical breeding programs, the genotyping strategy should be designed so that both live and dead animals are genotyped. Ideally, all animals should be systematically genotyped at birth, such that the dataset would contain genomic information from both live animals and those that die after birth. Since genotyping all animals is costly, selective genotyping can be a more cost-effective strategy. To implement this, tissue samples could be collected at birth on all animals and stored. Tissues can be collected from the tail or the ear or a few mL of blood can be sampled from the tail for genotyping depending on the company’s procedures. Then, when an animal dies, the stored tissue sample can be sent to the genotyping company. To implement this, it is very important that all animals are tagged properly when collecting samples.

Genotyping only live animals resulted in much greater genetic gain compared to pedigree-based selection. In pedigree-based selection, it is difficult to differentiate genetically superior animals among the survivors based on pedigree information and parent–offspring phenotyping combinations. However, genotyping only live animals may not overcome the limitation in pedigree-based selection because there still is no contrast in the phenotype of the genotyped animals. Nevertheless, the simulations based on genotyping different proportions of live animals clearly showed that genotyping live animals resulted in much greater genetic gain for PWS compared to pedigree-based selection. The benefits of genotyping live animals only compared to pedigree-based selection can be explained by the use of the single step method to predict breeding values for PWS using phenotypes of both live and dead animals and the genotypes of live animals. The **H** matrix includes relationships between genotyped live animals and non-genotyped dead animals [18]. Thus, based on this matrix and the contrasting phenotypes of both live and dead animals, genotyping only live animals realised a 26 to 52% greater genetic gain compared to pedigree-based selection, but genotyping both live and dead animals resulted in even greater genetic gain than genotyping live animals only.

Our results show that genomic predictions reduced the deviation of bias from 1 compared to pedigree-based selection. Many factors can cause bias in genetic evaluations, including selective phenotyping, use of highly selected animals in genetic analysis, selective reporting of phenotypes, and inappropriate statistical models [26]. Some of these factors can cause bias if they change the basic assumption of a null expectation of Mendelian sampling terms [26]. In our study, the difference in bias of EBV resulting from genotyping both live and dead animals and genotyping only live animals was small. This gives more incentives to use single-step genetic evaluation for unbiased prediction because single-step genomic prediction accounts for bias from selective genotyping [7]. Furthermore, we estimated the variance components using a pedigree-based model and used them in the single-step genomic evaluation for all genotyping strategies, which also helped to reduce bias [9]. The estimated bias of the EBV for PWS in our study was smaller than that reported for mortality by Gebreyesus et al. [17] who used a threshold model, which could be due to different methods for transforming EBVs to probabilities in the study by Gebreyesus et al. [17]. Although all the animals with extreme phenotypes were genotyped in our study, the regression coefficients of TBV on EBV still deviated from 1, which is in agreement with other studies such as Odegard and Meuwissen [25] and Gowane et al. [7]. However, Odegard and Meuwissen [25] suggested that a bias of small magnitude would not be problematic in reality.

In the current study, both the linear and the threshold models were used to predict EBV for PWS, and similar accuracies of the EBV were found. For practical application, the linear model is advantageous because it can easily be extended for joint evaluation of several breeding objective traits. Indeed, multitrait animal models are useful in practice since, in animal breeding programs, it is interesting to determine the total merit of selection candidates [27].

This study was conducted using a single trait for selection, with selection pressure fully acting on PWS. However, practical pig breeding programs include multiple traits for simultaneous improvement of all traits [28]. As more traits are included in the breeding objective, the relative selection pressure for each trait decreases compared to single trait selection. If the current study had applied multi-trait selection, the genetic gain in PWS would have been smaller than that obtained here. In addition, a very simple breeding program was simulated in this study, i.e. absence of noise due to the addition of different fixed effects in the model to predict breeding values for PWS. Only different population sizes, and therefore different selection intensities, were used to test the hypothesis and the hypothesis turned out to be true in different population sizes. In addition, we did not investigate the impact of truncation selection versus OCS on the rate of genetic gain for PWS. Both the heritability and additive genetic variance for PWS were low. In case of a phenotype with a low heritability, sib information becomes more important [29], which indicates that realising a higher rate of genetic gain for a lowly heritable trait can increase co-selection of relatives using truncation selection, which, in turn, can increase the rate of inbreeding [29]. Therefore, OCS was used with a rate of inbreeding restricted to 1% pedigree-based inbreeding per generation. However, the conclusion inferred from this study would be same for multi-trait truncation selection and complex breeding programs, i.e. that genotyping both live and dead animals is expected to result in greater genetic gain for PWS compared to genotyping only live animals.

## Conclusions

In this study, we simulated breeding schemes with different rates of post-weaning survival and litter sizes to investigate the impact of genotyping both live and dead animals on the genetic gain for post-weaning survival. The results showed that genotyping both live and dead animals resulted in 14 to 33% more genetic gain for post-weaning survival than genotyping only live animals in all combinations of breeding schemes using both the linear model and the threshold model. We also found that genotyping only 60% of dead animals along with genotyping live animals resulted in most of the genetic gain compared to genotyping all live and dead animals. Genotyping both live and dead animals increased the accuracy of estimated breeding values for post-weaning survival and this increased prediction accuracy translated into increased genetic gain. We conclude that the genotyping strategy for the genetic improvement of post-weaning survival in pigs should include the genomic information from both live and dead animals in genomic selection.

## Data Availability

The simulated data of this study are available from the corresponding author upon reasonable request.
